# Advances in genetic hearing loss: *CIB2* gene

**DOI:** 10.1007/s00405-016-4330-9

**Published:** 2016-10-22

**Authors:** Agnieszka Jacoszek, Agnieszka Pollak, Rafał Płoski, Monika Ołdak

**Affiliations:** 1 Department of Medical Genetics, Warsaw Medical University, Warsaw, Poland; 2 Postgraduate School of Molecular Medicine, Warsaw, Poland; 3 Department of Genetics, Institute of Physiology and Pathology of Hearing, Mokra 17, Nadarzyn, Warsaw/Kajetany, 05-830 Poland

**Keywords:** Hearing loss, Nonsyndromic, DFNB48, Usher syndrome, *CIB2*, Calcium, Integrin

## Abstract

Hearing plays a crucial role in human development. Receiving and processing sounds are essential for the advancement of the speech ability during the early childhood and for a proper functioning in the society. Hearing loss is one of the most frequent disabilities that affect human senses. It can be caused by genetic or environmental factors or both of them. Calcium- and integrin-binding protein 2 (CIB2) is one of the recently identified genes, involved in HI pathogenesis. *CIB2* is widely expressed in various human and animal tissues, mainly in skeletal muscle, nervous tissue, inner ear, and retina. The CIB2 protein is responsible for maintaining Ca^2+^ homeostasis in cells and interacting with integrins—transmembrane receptors essential for cell adhesion, migration, and activation of signaling pathways. Calcium signaling pathway is crucial for signal transduction in the inner ear, and integrins regulate hair cell differentiation and maturation of the stereocilia. To date, mutations detected in *CIB2* are causative for nonsyndromic hearing loss (DFNB48) or Usher syndrome type 1 J. Patients harboring biallelic *CIB2* mutations suffer from bilateral, early onset, moderate to profound HI. In the paper, we summarize the current status of the research on *CIB2*.

## Introduction

Receiving and processing sounds are essential for a proper development of communication skills during the early childhood. Hearing loss is one of the most frequent disabilities that affect human senses. According to the World Health Organization [1], over 5 % of world population (360 million of people, including 32 million of children) suffers from hearing impairment (HI). This term refers to hearing loss greater than 40 decibels (dB) in the better hearing ear in adults and 30 dB in children. The prevalence of newborns with HI is estimated to 2–4 per 1000 in the developed countries and 6 per 1000 in the developing countries [2]. Apart from environmental factors, such as noise, infections, and ototoxic drugs [3]. HI can be caused by genetic factors or a combination of both of them.

More than a half (50–60 %) of the congenital hearing loss cases are due to genetic factors. To date, approximately 300 genes are considered related to the process of hearing [4]. Most of them encode proteins involved in the structure and function of inner ear.

Recently, *CIB2* (calcium- and integrin-binding protein 2) gene has been added to the extensive list of genes associated with hearing, loss [5].

## *CIB2* general information

**Fig. 1:**
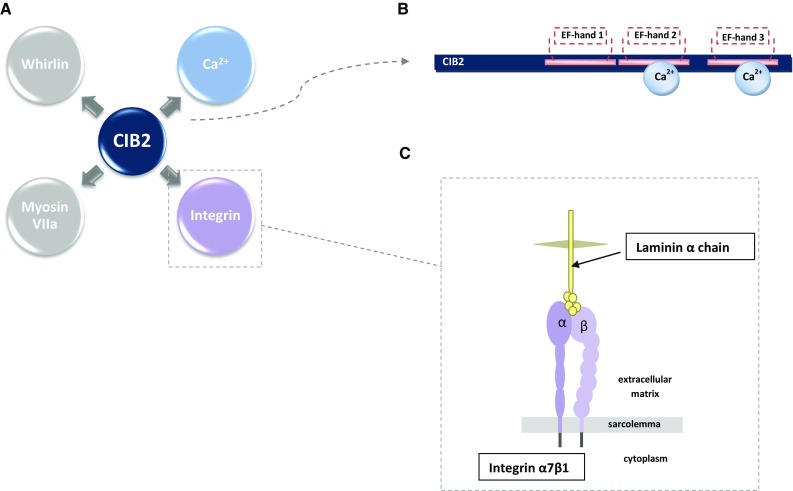
Multiple roles of CIB2 protein. **a** Scheme of the interactions of CIB2 protein with other molecules. **b** CIB2 binds Ca^2+^ ions through the second and third EF-hand domains. **c** Integrin α7β1 is a heterodimeric transmembrane receptor for laminin

The *CIB2* gene (MIM# 605564) is localized on chromosome 15 (15q25.1) [6], encodes four different isoforms that consist of 4–6 exons [5]. CIB2 protein plays a role in calcium ions homeostasis and interacts with integrins (Fig. 1a) [7]. Ca^2+^ is a crucial molecule in cellular signaling pathways and also takes part in signal transduction in the inner ear in the organ of Corti. It is one of the factors determining the transmission of sound and balance information through the hair cell apical mechanosensitive transduction (MET) channels to the ribbon synapse at the bottom of the hair cells. MET channels participate in the transport of potassium and calcium ions from the endolymph to the hair cells. Ca^2+^ is a second messenger that induces conformational changes in effector molecules regulating the sensitivity of the cochlear amplifier, which is a mechanism of increasing the amplitude and frequency selectivity of sound waves. Consequently, the intracellular Ca^2+^ concentration is crucial for MET channels adaptation, frequency tuning, hair bundle twitching, outer hair cells (OHC), electromotility, and afferent synaptic transmission [5, 8–10].

Integrins are essential for cell adhesion and activation of intra- and extracellular signaling pathways. They are α/β heterodimeric transmembrane receptors for ligands in the extracellular matrix (Fig. 1b). Integrins and their ligands play key roles in the development as well as in different cellular functions, such as immune responses or hemostasis. Their dysfunction may be the cause of many diseases, which makes them a promising target for the rapidly developing, effective therapies against, e.g., thrombosis and inflammation [11, 12]. In the sound transduction system, several integrins control the process of stereocilia maturation and hair cell differentiation [13]. Furthermore, integrins regulate the dynamics of actin, thereby they determine the proper function of the F-actin cytoskeleton—an important structure of the sensory hair cells [14]. Thus, integrins are listed in the plethora of the indispensable elements of the auditory pathway.

## *CIB2* pattern of expression

Studies on the *CIB* gene family started in the 1990s when two independent research groups found a novel protein that was named according to its putative function, i.e., CIB (calcium-binding protein) [15] or KIP (kinase interacting protein) [16], respectively. These first findings demonstrated that the CIB/KIP protein shares sequence identity with calmodulin and calcineurin B, interacted with nuclear DNA-dependent serine/threonine protein kinase, and presented a calcium and integrin binding activity [6, 15–17]. The same protein was also known as calmyrin due to its ability to bind Ca^2+^ and undergo N-myristoylation [18]. The latter is a process of attachment of a 14-carbon saturated fatty acid, myristate, to the N-terminal glycine residue of specific target proteins in the cell, which may influence intra- and intercellular interactions [19–22]. Except for *CIB2*, the human genome contains three other genes (*CIB1*, *CIB3*, and *CIB4*) encoding highly homologous proteins, which together form a family of calcium-binding proteins and contain elongation factor-hand (EF-hand) domains [23].

Nonquantitative reverse transcription PCR analysis of human tissues showed that *CIB2* mRNA was transcribed in a wide variety of human tissues, including brain, lungs, skeletal muscles [6], and human platelets [15], but so far, no data have been published on the expression pattern of *CIB2* in the human ear or retina. Further studies in mice confirmed that expression of *Cib2* included also the inner ear (cochlea) and retina [5]. Particular localization of this protein was established in animal studies, mainly in mice due to the fact that mouse is a key model organism in analyzing mammalian developmental, physiological, and disease processes in hearing impairment [24]. When extrapolating the results of mouse or rat studies to human, it should be emphasized that in rodents, the majority of hair cells and supporting cells are formed during the embryonic development, but the organ of Corti undergoes processes of development and maturation also after birth [25–27]. It is unlike in human, when this unit is fully developed at the moment of birth [28].

Transcripts of the *Cib2* gene are present in embryos, throughout postnatal development as well as in adult mice [17]. Using in situ hybridization high level of *Cib2* expression was observed in forebrain, midbrain, hindbrain, spinal cord, somites, inner ear, vibrissae, gut, and musculature in mouse embryos. In the ear, *Cib2* transcripts were detected in the cytoplasm of adult supporting cells, inner hair cells, OHC, cuticular plate (an apical cytoplasm of the hair cell formed of actin filaments), and along the stereocilia. The signal for Cib2 was usually more intense in the shorter row of stereocilia tips than in the longer row. The transcript was first observed at postnatal day 2 in the developing organ of Corti and vestibular organs. Until postnatal day 8, it was limited to supporting cells in the organ of Corti. In the retina, Cib2 was localized in inner and outer segments of photoreceptor cells and in retinal pigment epithelium. A signal of diffused immunoreactivity was also detected in the inner plexiform layers, outer plexiform layers, and the ganglion cell layer [5]. Semiquantitative and quantitative real-time PCR of skeletal muscle, liver, brain, spleen, heart, kidney, and lung revealed that in adult mice, *Cib2* mRNA is mainly expressed in skeletal muscles. Within the muscle, *Cib2* is expressed in sarcolemma, enriched in the myotendinous junctions and neuromuscular junctions. Lower levels of expression were noticeable in brain and in lungs [7].

In the rat brain tissue, the *Cib2* transcript was observed in several areas and the highest level of expression was detected in the hippocampus (*cornu ammonis* area three regions and *dentate gyrus*). Furthermore, it was also found in sensory, entorhinal, and prefrontal cortex. Intracellular localization of the Cib2 protein in rats is considered to be the Golgi apparatus and in nerve cells the protein localizes mainly to neurites [18].

In sheep, the expression of *Cib2* mRNA was detected in many various tissues, mainly in stomach, heart, and ovary [29]. Expression of *Cib2* in zebrafish is detected throughout the development. *Drosophila* gene *CG9236*, encoding a protein which is in 71 % similar and in 59 % identical to the human *CIB2*, is expressed in several larval and imago tissues, therein the adult eye [5].

## CIB2 protein, its function, and interactions with other molecules

The crucial functional units of the CIB family proteins are the EF-hand domains, one of the most common structural protein motifs in mammalian cells [30], which are able to bind Ca^2+^ and Mg^2+^ ions. They are considered as regulatory motifs that mediate responses to changes in calcium concentration and fulfill a role of intracellular calcium signaling mediators. CIB2 contains three EF-hand domains and through the second and third domains, it is able to bind Ca^2+^ (Fig. 1b). The first EF-hand domain is not functional [18, 23, 31, 32]. EF-hand Ca^2+^ binding proteins have a crucial role in all aspects of Ca^2+^ signaling, having diverse roles that range from controlling the functioning of Ca^2+^ channels to moderating the intensity and duration of Ca^2+^ signals and transducing them into biochemical and biomechanical responses [33]. It is believed that EF-hand Ca^2+^ buffers regulate presynaptic inner hair cells function for metabolically efficient sound coding [34]. Upon binding Ca^2+^ ions, Cib2 changes its conformation into a Ca^2+^-bound form, which is one of the characteristic properties for proteins transmitting Ca^2+^ signals. Based on the localization of Cib2 in stereocilia, it can be hypothesized that CIB2 temporarily captures calcium entering the stereocilia through MET channels until the ions exit stereocilia through the plasma membrane Ca^2+^ ATPase or are uptaken by mitochondria. This is consistent with the speculations that Cib2 may be involved in calcium signaling that regulates MET in the inner ear [18, 35].

The CIB2 protein binds to myosin VIIa and whirlin (Fig. 1a), which makes it a part of the usher syndrome interactome, but none of the protein is required for proper localization of Cib2 in the mouse stereocilia [5]. To date, there is no published information on particular mechanisms following this interaction.

The presence of CIB2 in skeletal muscle and nervous tissue suggests that Cib2 can play a significant role in the development of the central nervous system and musculature. Laminin 2 is a protein required for muscle development and stability and the laminin α2 chain is absent in mice with severe muscular dystrophy [36]. In muscle cells, integrin α7β1 is one of the major laminin α2 chain-binding receptors (Fig. 1c) [37], and it is responsible for proper muscle function. Studies on muscular dystrophy type 1A mouse showed that in laminin α2 chain-deficient muscle, not only integrin α7B subunit but also *Cib2* expression was reduced. It seems to be a consequence of decreased integrin α7B level and supports the hypothesis of direct integrin α7B and CIB2 interaction. These findings are consistent with co-expression of Cib2 with integrin α7β subunit in skeletal muscle and embryonic nervous system [7].

## *CIB2* gene mutations

To date, seven mutations in the *CIB2* gene (RefSeq: NM_006383.2, NP_006374.1) have been discovered: c.97C>T (p.Arg33*), c.192G>C (p.Glu64Asp), c.196C>T (p.Arg66Trp), c.272T>C (p.Phe91Ser), c.297C>G (p.Cys99Trp), c.368T>C (p.Ile123Thr), and c.556C>T (p.Arg186Trp) [5, 38, 39]. All of them except c.97C>T and c.556C>T affect the three of four alternatively spliced isoforms, i.e., A, B, and C, of the CIB2 protein. Mutation c.97C>T affects isoforms B and C, but not the isoforms A and CIB2-006 [5, 39], while c.556C>T affects presumably [38], isoforms A, B, and CIB2-006. As all the identified *CIB2* mutations can be assigned to isoform B, it indicates that this particular isoform is the most significant one for the process of sound transduction, but further research is necessary to confirm the presumption.

To date, all mutations discovered in the *CIB2* gene present a recessive pattern of inheritance. The majority of them lead to hearing loss (DFNB48) and only p.Glu64Asp was identified in a family with Usher syndrome type 1 J. All individuals harboring homozygous or compound heterozygous *CIB2* variants suffered from the early onset, bilateral, moderate to profound HI [5, 38, 39]. Although primarily examined patients with *CIB2* mutations present Pakistani and Turkish origin, further research revealed also Dutch and Caribbean Hispanic *CIB2* families. It indicates that individuals originating from other than Pakistani and Turkish populations may carry mutations in the *CIB2* gene [5, 38, 39].

The c.192G>C, c.272T>C, and c.297C>G variants probably reduce the interaction of CIB2 with integrins and modify their activation. The c.192G>C mutation changes the protein conformation, thereby it affects binding affinity or kinetics of integrin. All these amino acids substitutions may cause slight changes in subcellular location of the protein which possibly affects the efficiency of calcium sequestration [5]. Nevertheless, Seco et al. provided further data for the c.272T>C mutation and suggest that this variant does not influence calcium-buffering abilities of *CIB2* [39]. The c.272T>C as well as c.297C>G mutations may disrupt effector binding site or Ca^2+^ binding by the second EF-hand domain. On the contrary, the c.368T>C variant probably magnifies the affinity of Ca^2+^ binding. None of the mutations mentioned above induce significant changes in CIB2 distribution in tissues [5]. The c.556C>T mutation (the most C-terminal localized one) affects neither the tip localization of CIB2 nor its interaction with whirlin but impairs the calcium-binding properties [38]. Variants c.196C>T and c.97C>T, as well as c.272T>C, do not affect ATP-induced calcium responses in cells, but probably alter integrin binding. Moreover, the c.97C>T variant may result in nonsense-mediated decay (degradation of the aberrant mRNAs harboring premature termination codon) [39, 40].

Different disorders other than HI or Usher syndrome have also been linked to the 15q24 locus, containing the *CIB2* gene. The deletion of this region was reported in patients suffering from abnormalities of the ears (cleft earlobe, preauricular tags, cupped, and underdeveloped auricles) hypotonia and developmental delay. Linkage analysis in a Pakistani family with spasticity, severe mental retardation and visual impairment, pointed that the *CIB2* gene may be involved in the pathogenesis of these abnormalities [7, 41, 42]. The most recent study provides an evidence that increased *CIB2* expression may also play a protective role in cardiovascular diseases by decreasing the pace of the vascular calcification [43].

## Conclusion and future perspectives

Although some research has already been performed on the *CIB2* gene, its function still remains unclear and is to be fully discovered. Mutations in *CIB2,* which so far have been revealed, segregate with both DFNB48 and USH1 J. Thus, *CIB2* is a causative gene for both disorders. Nevertheless, further studies are required for a better understanding of the role of the *CIB2* gene in human. Clarification of its function and associated molecular mechanisms will be the next step towards better prevention and treatment of hearing loss or cardiovascular diseases in patients, thereby towards improved living standards of people at risk of CIB2-associated diseases.
